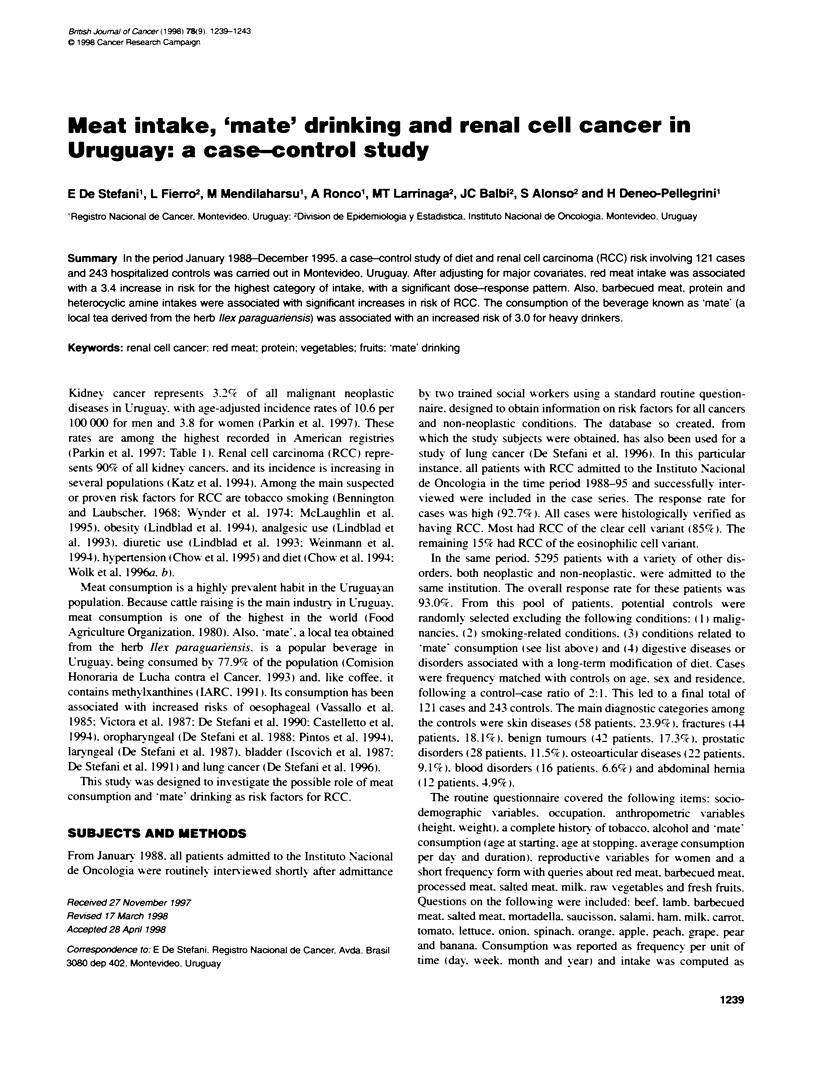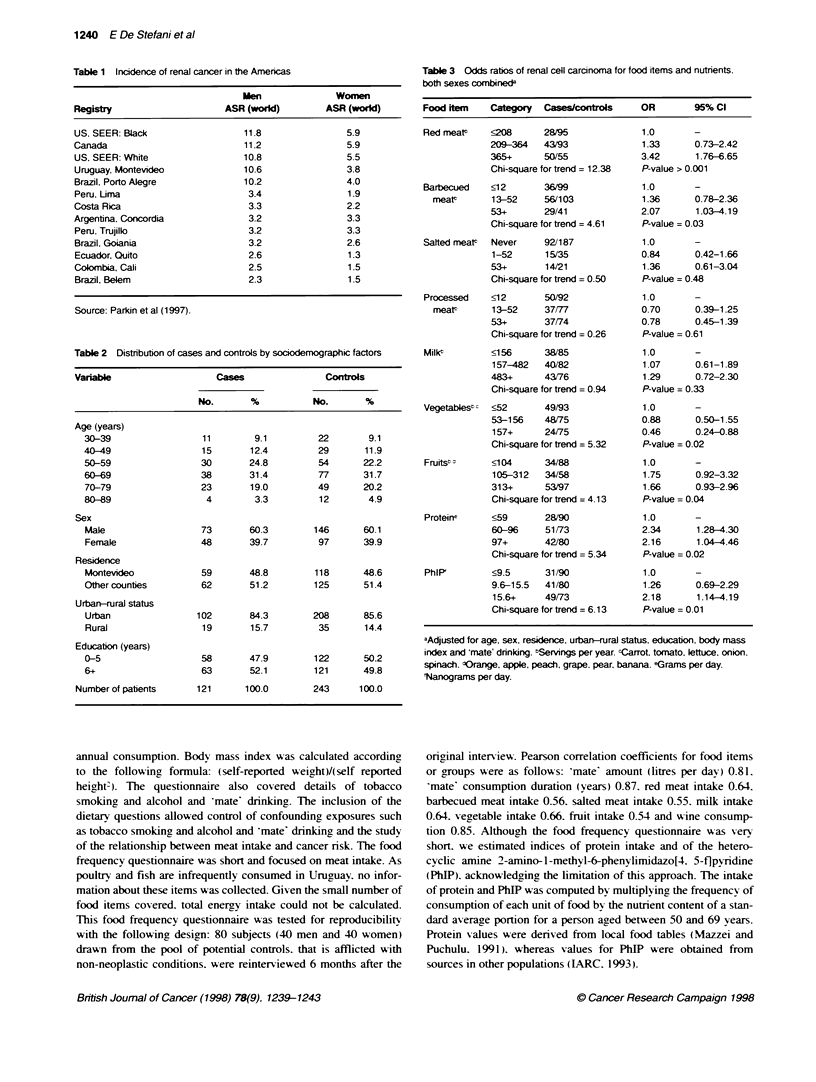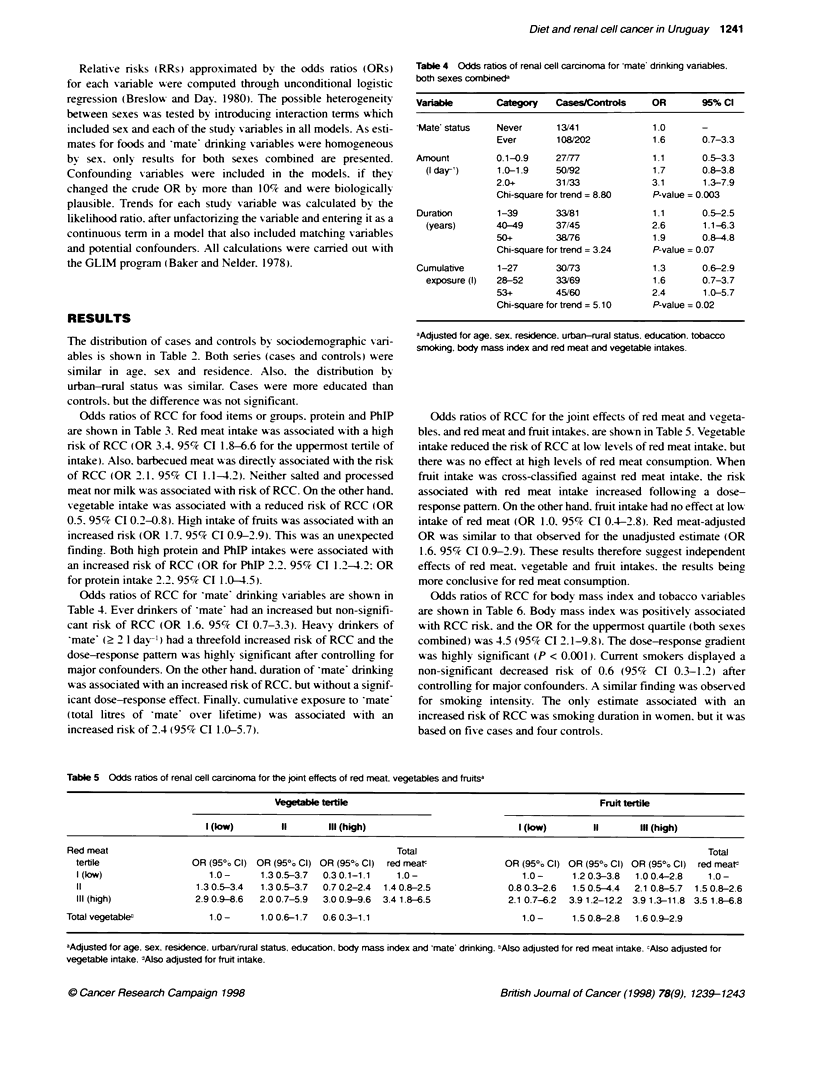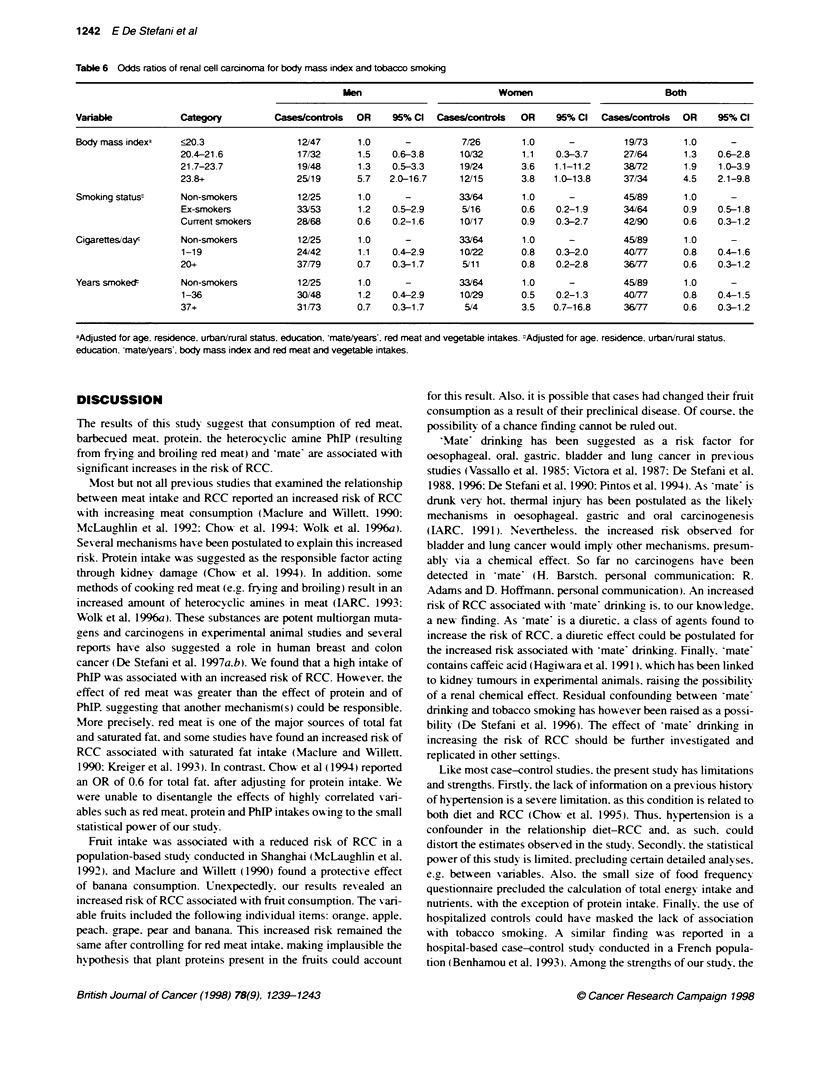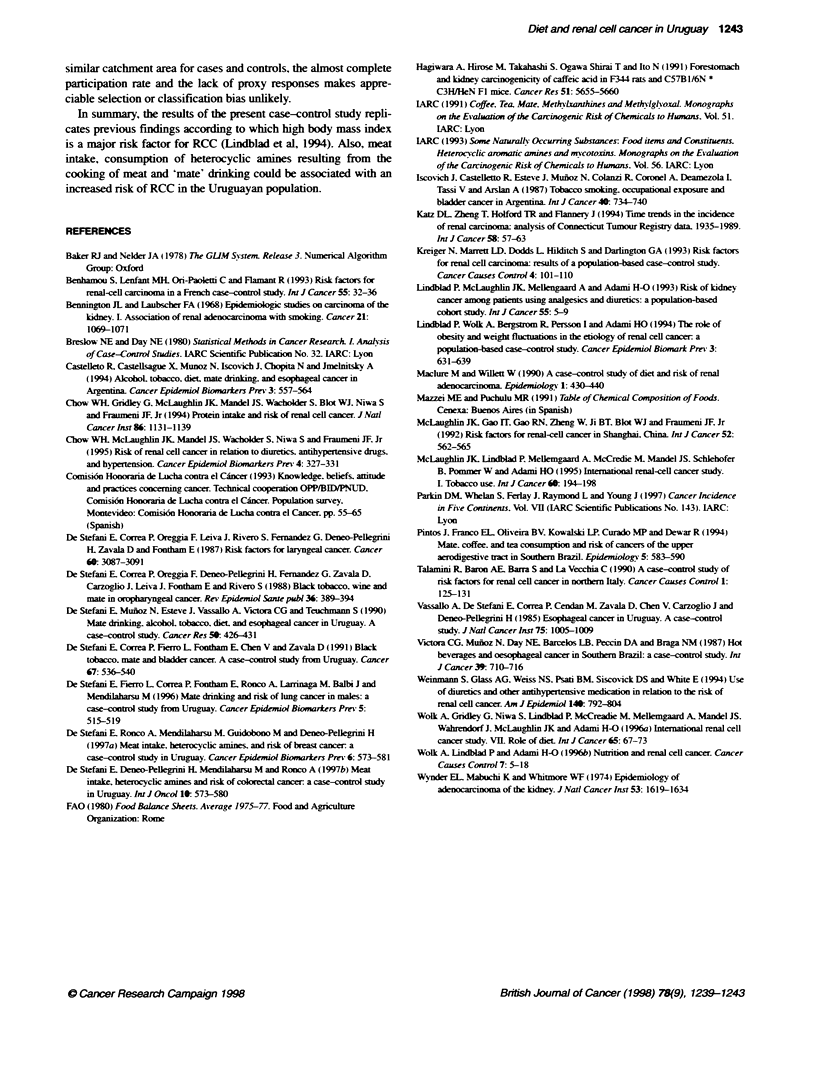# Meat intake, 'mate' drinking and renal cell cancer in Uruguay: a case-control study.

**DOI:** 10.1038/bjc.1998.661

**Published:** 1998-11

**Authors:** E. De Stefani, L. Fierro, M. Mendilaharsu, A. Ronco, M. T. Larrinaga, J. C. Balbi, S. Alonso, H. Deneo-Pellegrini

**Affiliations:** Registro Nacional de Cancer, Montevideo, Uruguay.

## Abstract

In the period January 1988-December 1995, a case-control study of diet and renal cell carcinoma (RCC) risk involving 121 cases and 243 hospitalized controls was carried out in Montevideo, Uruguay. After adjusting for major covariates, red meat intake was associated with a 3.4 increase in risk for the highest category of intake, with a significant dose-response pattern. Also, barbecued meat, protein and heterocyclic amine intakes were associated with significant increases in risk of RCC. The consumption of the beverage known as 'mate' (a ocal tea derived from the herb Ilex paraguariensis) was associated with an increased risk of 3.0 for heavy drinkers.


British Journal of Cancer (1998) 78(9), 1239–1243
© 1998 Cancer Research Campaign

### Meat intake, 'mate' drinking and renal cell cancer in Uruguay: a case–control study

E De Stefani[1], L Fierro[2], M Mendilaharsu[1], A Ronco[1], MT Larrinaga[2], JC Balbi[2], S Alonso[2] and H Deneo-Pellegrini[1]

[1]Registro Nacional de Cancer, Montevideo, Uruguay; [2]Division de Epidemiologia y Estadistica, Instituto Nacional de Oncologia, Montevideo, Uruguay

**Summary** In the period January 1988–December 1995, a case–control study of diet and renal cell carcinoma (RCC) risk involving 121 cases and 243 hospitalized controls was carried out in Montevideo, Uruguay. After adjusting for major covariates, red meat intake was associated with a 3.4 increase in risk for the highest category of intake, with a significant dose–response pattern. Also, barbecued meat, protein and heterocyclic amine intakes were associated with significant increases in risk of RCC. The consumption of the beverage known as 'mate' (a local tea derived from the herb *Ilex paraguariensis*) was associated with an increased risk of 3.0 for heavy drinkers.

**Keywords:** renal cell cancer; red meat; protein; vegetables; fruits; 'mate' drinking

Kidney cancer represents 3.2% of all malignant neoplastic diseases in Uruguay, with age-adjusted incidence rates of 10.6 per 100 000 for men and 3.8 for women (Parkin et al, 1997). These rates are among the highest recorded in American registries (Parkin et al, 1997; Table 1). Renal cell carcinoma (RCC) represents 90% of all kidney cancers, and its incidence is increasing in several populations (Katz et al, 1994). Among the main suspected or proven risk factors for RCC are tobacco smoking (Bennington and Laubscher, 1968; Wynder et al, 1974; McLaughlin et al, 1995), obesity (Lindblad et al, 1994), analgesic use (Lindblad et al, 1993), diuretic use (Lindblad et al, 1993; Weinmann et al, 1994), hypertension (Chow et al, 1995) and diet (Chow et al, 1994; Wolk et al, 1996*a*, *b*).

Meat consumption is a highly prevalent habit in the Uruguayan population. Because cattle raising is the main industry in Uruguay, meat consumption is one of the highest in the world (Food Agriculture Organization, 1980). Also, 'mate', a local tea obtained from the herb *Ilex paraguariensis*, is a popular beverage in Uruguay, being consumed by 77.9% of the population (Comision Honoraria de Lucha contra el Cancer, 1993) and, like coffee, it contains methylxanthines (IARC, 1991). Its consumption has been associated with increased risks of oesophageal (Vassallo et al, 1985; Victora et al, 1987; De Stefani et al, 1990; Castelletto et al, 1994), oropharyngeal (De Stefani et al, 1988; Pintos et al, 1994), laryngeal (De Stefani et al, 1987), bladder (Iscovich et al, 1987; De Stefani et al, 1991) and lung cancer (De Stefani et al, 1996).

This study was designed to investigate the possible role of meat consumption and 'mate' drinking as risk factors for RCC.

### SUBJECTS AND METHODS

From January 1988, all patients admitted to the Instituto Nacional de Oncologia were routinely interviewed shortly after admittance by two trained social workers using a standard routine questionnaire, designed to obtain information on risk factors for all cancers and non-neoplastic conditions. The database so created, from which the study subjects were obtained, has also been used for a study of lung cancer (De Stefani et al, 1996). In this particular instance, all patients with RCC admitted to the Instituto Nacional de Oncologia in the time period 1988–95 and successfully interviewed were included in the case series. The response rate for cases was high (92.7%). All cases were histologically verified as having RCC. Most had RCC of the clear cell variant (85%). The remaining 15% had RCC of the eosinophilic cell variant.

In the same period, 5295 patients with a variety of other disorders, both neoplastic and non-neoplastic, were admitted to the same institution. The overall response rate for these patients was 93.0%. From this pool of patients, potential controls were randomly selected excluding the following conditions: (1) malignancies, (2) smoking-related conditions, (3) conditions related to 'mate' consumption (see list above) and (4) digestive diseases or disorders associated with a long-term modification of diet. Cases were frequency matched with controls on age, sex and residence, following a control–case ratio of 2:1. This led to a final total of 121 cases and 243 controls. The main diagnostic categories among the controls were skin diseases (58 patients, 23.9%), fractures (44 patients, 18.1%), benign tumours (42 patients, 17.3%), prostatic disorders (28 patients, 11.5%), osteoarticular diseases (22 patients, 9.1%), blood disorders (16 patients, 6.6%) and abdominal hernia (12 patients, 4.9%).

The routine questionnaire covered the following items: sociodemographic variables, occupation, anthropometric variables (height, weight), a complete history of tobacco, alcohol and 'mate' consumption (age at starting, age at stopping, average consumption per day and duration), reproductive variables for women and a short frequency form with queries about red meat, barbecued meat, processed meat, salted meat, milk, raw vegetables and fresh fruits. Questions on the following were included: beef, lamb, barbecued meat, salted meat, mortadella, saucisson, salami, ham, milk, carrot, tomato, lettuce, onion, spinach, orange, apple, peach, grape, pear and banana. Consumption was reported as frequency per unit of time (day, week, month and year) and intake was computed as

*Received 27 November 1997*
*Revised 17 March 1998*
*Accepted 28 April 1998*

*Correspondence to:* E De Stefani, Registro Nacional de Cancer, Avda. Brasil 3080 dep 402, Montevideo, Uruguay

Table 1 Incidence of renal cancer in the Americas

| Registry | Men ASR (world) | Women ASR (world) |
|---|---|---|
| US, SEER: Black | 11.8 | 5.9 |
| Canada | 11.2 | 5.9 |
| US, SEER: White | 10.8 | 5.5 |
| Uruguay, Montevideo | 10.6 | 3.8 |
| Brazil, Porto Alegre | 10.2 | 4.0 |
| Peru, Lima | 3.4 | 1.9 |
| Costa Rica | 3.3 | 2.2 |
| Argentina, Concordia | 3.2 | 3.3 |
| Peru, Trujillo | 3.2 | 3.3 |
| Brazil, Goiania | 3.2 | 2.6 |
| Ecuador, Quito | 2.6 | 1.3 |
| Colombia, Cali | 2.5 | 1.5 |
| Brazil, Belem | 2.3 | 1.5 |

Source: Parkin et al (1997).

Table 2 Distribution of cases and controls by sociodemographic factors

| Variable | Cases | | Controls | |
|---|---|---|---|---|
| | No. | % | No. | % |
| Age (years) | | | | |
| 30–39 | 11 | 9.1 | 22 | 9.1 |
| 40–49 | 15 | 12.4 | 29 | 11.9 |
| 50–59 | 30 | 24.8 | 54 | 22.2 |
| 60–69 | 38 | 31.4 | 77 | 31.7 |
| 70–79 | 23 | 19.0 | 49 | 20.2 |
| 80–89 | 4 | 3.3 | 12 | 4.9 |
| Sex | | | | |
| Male | 73 | 60.3 | 146 | 60.1 |
| Female | 48 | 39.7 | 97 | 39.9 |
| Residence | | | | |
| Montevideo | 59 | 48.8 | 118 | 48.6 |
| Other counties | 62 | 51.2 | 125 | 51.4 |
| Urban–rural status | | | | |
| Urban | 102 | 84.3 | 208 | 85.6 |
| Rural | 19 | 15.7 | 35 | 14.4 |
| Education (years) | | | | |
| 0–5 | 58 | 47.9 | 122 | 50.2 |
| 6+ | 63 | 52.1 | 121 | 49.8 |
| Number of patients | 121 | 100.0 | 243 | 100.0 |

Table 3 Odds ratios of renal cell carcinoma for food items and nutrients, both sexes combined[a]

| Food item | Category | Cases/controls | OR | 95% CI |
|---|---|---|---|---|
| Red meat[b] | ≤208 | 28/95 | 1.0 | – |
| | 209–364 | 43/93 | 1.33 | 0.73–2.42 |
| | 365+ | 50/55 | 3.42 | 1.76–6.65 |
| | Chi-square for trend = 12.38 | | *P*-value > 0.001 | |
| Barbecued meat[b] | ≤12 | 36/99 | 1.0 | – |
| | 13–52 | 56/103 | 1.36 | 0.78–2.36 |
| | 53+ | 29/41 | 2.07 | 1.03–4.19 |
| | Chi-square for trend = 4.61 | | *P*-value = 0.03 | |
| Salted meat[b] | Never | 92/187 | 1.0 | – |
| | 1–52 | 15/35 | 0.84 | 0.42–1.66 |
| | 53+ | 14/21 | 1.36 | 0.61–3.04 |
| | Chi-square for trend = 0.50 | | *P*-value = 0.48 | |
| Processed meat[b] | ≤12 | 50/92 | 1.0 | – |
| | 13–52 | 37/77 | 0.70 | 0.39–1.25 |
| | 53+ | 37/74 | 0.78 | 0.45–1.39 |
| | Chi-square for trend = 0.26 | | *P*-value = 0.61 | |
| Milk[b] | ≤156 | 38/85 | 1.0 | – |
| | 157–482 | 40/82 | 1.07 | 0.61–1.89 |
| | 483+ | 43/76 | 1.29 | 0.72–2.30 |
| | Chi-square for trend = 0.94 | | *P*-value = 0.33 | |
| Vegetables[b,c] | ≤52 | 49/93 | 1.0 | – |
| | 53–156 | 48/75 | 0.88 | 0.50–1.55 |
| | 157+ | 24/75 | 0.46 | 0.24–0.88 |
| | Chi-square for trend = 5.32 | | *P*-value = 0.02 | |
| Fruits[b,d] | ≤104 | 34/88 | 1.0 | – |
| | 105–312 | 34/58 | 1.75 | 0.92–3.32 |
| | 313+ | 53/97 | 1.66 | 0.93–2.96 |
| | Chi-square for trend = 4.13 | | *P*-value = 0.04 | |
| Protein[e] | ≤59 | 28/90 | 1.0 | – |
| | 60–96 | 51/73 | 2.34 | 1.28–4.30 |
| | 97+ | 42/80 | 2.16 | 1.04–4.46 |
| | Chi-square for trend = 5.34 | | *P*-value = 0.02 | |
| PhIP[f] | ≤9.5 | 31/90 | 1.0 | – |
| | 9.6–15.5 | 41/80 | 1.26 | 0.69–2.29 |
| | 15.6+ | 49/73 | 2.18 | 1.14–4.19 |
| | Chi-square for trend = 6.13 | | *P*-value = 0.01 | |

[a]Adjusted for age, sex, residence, urban–rural status, education, body mass index and 'mate' drinking. [b]Servings per year. [c]Carrot, tomato, lettuce, onion, spinach. [d]Orange, apple, peach, grape, pear, banana. [e]Grams per day. [f]Nanograms per day.

annual consumption. Body mass index was calculated according to the following formula: (self-reported weight)/(self reported height$^2$). The questionnaire also covered details of tobacco smoking and alcohol and 'mate' drinking. The inclusion of the dietary questions allowed control of confounding exposures such as tobacco smoking and alcohol and 'mate' drinking and the study of the relationship between meat intake and cancer risk. The food frequency questionnaire was short and focused on meat intake. As poultry and fish are infrequently consumed in Uruguay, no information about these items was collected. Given the small number of food items covered, total energy intake could not be calculated. This food frequency questionnaire was tested for reproducibility with the following design: 80 subjects (40 men and 40 women) drawn from the pool of potential controls, that is afflicted with non-neoplastic conditions, were reinterviewed 6 months after the original interview. Pearson correlation coefficients for food items or groups were as follows: 'mate' amount (litres per day) 0.81, 'mate' consumption duration (years) 0.87, red meat intake 0.64, barbecued meat intake 0.56, salted meat intake 0.55, milk intake 0.64, vegetable intake 0.66, fruit intake 0.54 and wine consumption 0.85. Although the food frequency questionnaire was very short, we estimated indices of protein intake and of the heterocyclic amine 2-amino-1-methyl-6-phenylimidazo[4, 5-f]pyridine (PhIP), acknowledging the limitation of this approach. The intake of protein and PhIP was computed by multiplying the frequency of consumption of each unit of food by the nutrient content of a standard average portion for a person aged between 50 and 69 years. Protein values were derived from local food tables (Mazzei and Puchulu, 1991), whereas values for PhIP were obtained from sources in other populations (IARC, 1993).

© Cancer Research Campaign 1998

Relative risks (RRs) approximated by the odds ratios (ORs) for each variable were computed through unconditional logistic regression (Breslow and Day, 1980). The possible heterogeneity between sexes was tested by introducing interaction terms which included sex and each of the study variables in all models. As estimates for foods and 'mate' drinking variables were homogeneous by sex, only results for both sexes combined are presented. Confounding variables were included in the models, if they changed the crude OR by more than 10% and were biologically plausible. Trends for each study variable was calculated by the likelihood ratio, after unfactorizing the variable and entering it as a continuous term in a model that also included matching variables and potential confounders. All calculations were carried out with the GLIM program (Baker and Nelder, 1978).

### RESULTS

The distribution of cases and controls by sociodemographic variables is shown in Table 2. Both series (cases and controls) were similar in age, sex and residence. Also, the distribution by urban–rural status was similar. Cases were more educated than controls, but the difference was not significant.

Odds ratios of RCC for food items or groups, protein and PhIP are shown in Table 3. Red meat intake was associated with a high risk of RCC (OR 3.4, 95% CI 1.8–6.6 for the uppermost tertile of intake). Also, barbecued meat was directly associated with the risk of RCC (OR 2.1, 95% CI 1.1–4.2). Neither salted and processed meat nor milk was associated with risk of RCC. On the other hand, vegetable intake was associated with a reduced risk of RCC (OR 0.5, 95% CI 0.2–0.8). High intake of fruits was associated with an increased risk (OR 1.7, 95% CI 0.9–2.9). This was an unexpected finding. Both high protein and PhIP intakes were associated with an increased risk of RCC (OR for PhIP 2.2, 95% CI 1.2–4.2; OR for protein intake 2.2, 95% CI 1.0–4.5).

Odds ratios of RCC for 'mate' drinking variables are shown in Table 4. Ever drinkers of 'mate' had an increased but non-significant risk of RCC (OR 1.6, 95% CI 0.7–3.3). Heavy drinkers of 'mate' (≥ 2 l $day^{-1}$) had a threefold increased risk of RCC and the dose–response pattern was highly significant after controlling for major confounders. On the other hand, duration of 'mate' drinking was associated with an increased risk of RCC, but without a significant dose–response effect. Finally, cumulative exposure to 'mate' (total litres of 'mate' over lifetime) was associated with an increased risk of 2.4 (95% CI 1.0–5.7).

**Table 4** Odds ratios of renal cell carcinoma for 'mate' drinking variables, both sexes combined[a]

| Variable | Category | Cases/Controls | OR | 95% CI |
|---|---|---|---|---|
| 'Mate' status | Never | 13/41 | 1.0 | – |
| | Ever | 108/202 | 1.6 | 0.7–3.3 |
| Amount (l $day^{-1}$) | 0.1–0.9 | 27/77 | 1.1 | 0.5–3.3 |
| | 1.0–1.9 | 50/92 | 1.7 | 0.8–3.8 |
| | 2.0+ | 31/33 | 3.1 | 1.3–7.9 |
| | Chi-square for trend = 8.80 | | *P*-value = 0.003 | |
| Duration (years) | 1–39 | 33/81 | 1.1 | 0.5–2.5 |
| | 40–49 | 37/45 | 2.6 | 1.1–6.3 |
| | 50+ | 38/76 | 1.9 | 0.8–4.8 |
| | Chi-square for trend = 3.24 | | *P*-value = 0.07 | |
| Cumulative exposure (l) | 1–27 | 30/73 | 1.3 | 0.6–2.9 |
| | 28–52 | 33/69 | 1.6 | 0.7–3.7 |
| | 53+ | 45/60 | 2.4 | 1.0–5.7 |
| | Chi-square for trend = 5.10 | | *P*-value = 0.02 | |

[a]Adjusted for age, sex, residence, urban–rural status, education, tobacco smoking, body mass index and red meat and vegetable intakes.

Odds ratios of RCC for the joint effects of red meat and vegetables, and red meat and fruit intakes, are shown in Table 5. Vegetable intake reduced the risk of RCC at low levels of red meat intake, but there was no effect at high levels of red meat consumption. When fruit intake was cross-classified against red meat intake, the risk associated with red meat intake increased following a dose–response pattern. On the other hand, fruit intake had no effect at low intake of red meat (OR 1.0, 95% CI 0.4–2.8). Red meat-adjusted OR was similar to that observed for the unadjusted estimate (OR 1.6, 95% CI 0.9–2.9). These results therefore suggest independent effects of red meat, vegetable and fruit intakes, the results being more conclusive for red meat consumption.

Odds ratios of RCC for body mass index and tobacco variables are shown in Table 6. Body mass index was positively associated with RCC risk, and the OR for the uppermost quartile (both sexes combined) was 4.5 (95% CI 2.1–9.8). The dose–response gradient was highly significant ($P < 0.001$). Current smokers displayed a non-significant decreased risk of 0.6 (95% CI 0.3–1.2) after controlling for major confounders. A similar finding was observed for smoking intensity. The only estimate associated with an increased risk of RCC was smoking duration in women, but it was based on five cases and four controls.

**Table 5** Odds ratios of renal cell carcinoma for the joint effects of red meat, vegetables and fruits[a]

| | Vegetable tertile | | | | Fruit tertile | | | |
|---|---|---|---|---|---|---|---|---|
| | I (low) | II | III (high) | | I (low) | II | III (high) | |
| Red meat tertile | OR (95% CI) | OR (95% CI) | OR (95% CI) | Total red meat[c] | OR (95% CI) | OR (95% CI) | OR (95% CI) | Total red meat[d] |
| I (low) | 1.0 – | 1.3 0.5–3.7 | 0.3 0.1–1.1 | 1.0 – | 1.0 – | 1.2 0.3–3.8 | 1.0 0.4–2.8 | 1.0 – |
| II | 1.3 0.5–3.4 | 1.3 0.5–3.7 | 0.7 0.2–2.4 | 1.4 0.8–2.5 | 0.8 0.3–2.6 | 1.5 0.5–4.4 | 2.1 0.8–5.7 | 1.5 0.8–2.6 |
| III (high) | 2.9 0.9–8.6 | 2.0 0.7–5.9 | 3.0 0.9–9.6 | 3.4 1.8–6.5 | 2.1 0.7–6.2 | 3.9 1.2–12.2 | 3.9 1.3–11.8 | 3.5 1.8–6.8 |
| Total vegetable[b] | 1.0 – | 1.0 0.6–1.7 | 0.6 0.3–1.1 | | 1.0 – | 1.5 0.8–2.8 | 1.6 0.9–2.9 | |

[a]Adjusted for age, sex, residence, urban/rural status, education, body mass index and 'mate' drinking. [b]Also adjusted for red meat intake. [c]Also adjusted for vegetable intake. [d]Also adjusted for fruit intake.

**Table 6** Odds ratios of renal cell carcinoma for body mass index and tobacco smoking

| | | Men | | | Women | | | Both | | |
|---|---|---|---|---|---|---|---|---|---|---|
| **Variable** | **Category** | **Cases/controls** | **OR** | **95% CI** | **Cases/controls** | **OR** | **95% CI** | **Cases/controls** | **OR** | **95% CI** |
| Body mass index[a] | ≤20.3 | 12/47 | 1.0 | – | 7/26 | 1.0 | – | 19/73 | 1.0 | – |
| | 20.4–21.6 | 17/32 | 1.5 | 0.6–3.8 | 10/32 | 1.1 | 0.3–3.7 | 27/64 | 1.3 | 0.6–2.8 |
| | 21.7–23.7 | 19/48 | 1.3 | 0.5–3.3 | 19/24 | 3.6 | 1.1–11.2 | 38/72 | 1.9 | 1.0–3.9 |
| | 23.8+ | 25/19 | 5.7 | 2.0–16.7 | 12/15 | 3.8 | 1.0–13.8 | 37/34 | 4.5 | 2.1–9.8 |
| Smoking status[c] | Non-smokers | 12/25 | 1.0 | – | 33/64 | 1.0 | – | 45/89 | 1.0 | – |
| | Ex-smokers | 33/53 | 1.2 | 0.5–2.9 | 5/16 | 0.6 | 0.2–1.9 | 34/64 | 0.9 | 0.5–1.8 |
| | Current smokers | 28/68 | 0.6 | 0.2–1.6 | 10/17 | 0.9 | 0.3–2.7 | 42/90 | 0.6 | 0.3–1.2 |
| Cigarettes/day[c] | Non-smokers | 12/25 | 1.0 | – | 33/64 | 1.0 | – | 45/89 | 1.0 | – |
| | 1–19 | 24/42 | 1.1 | 0.4–2.9 | 10/22 | 0.8 | 0.3–2.0 | 40/77 | 0.8 | 0.4–1.6 |
| | 20+ | 37/79 | 0.7 | 0.3–1.7 | 5/11 | 0.8 | 0.2–2.8 | 36/77 | 0.6 | 0.3–1.2 |
| Years smoked[c] | Non-smokers | 12/25 | 1.0 | – | 33/64 | 1.0 | – | 45/89 | 1.0 | – |
| | 1–36 | 30/48 | 1.2 | 0.4–2.9 | 10/29 | 0.5 | 0.2–1.3 | 40/77 | 0.8 | 0.4–1.5 |
| | 37+ | 31/73 | 0.7 | 0.3–1.7 | 5/4 | 3.5 | 0.7–16.8 | 36/77 | 0.6 | 0.3–1.2 |

[a]Adjusted for age, residence, urban/rural status, education, 'mate/years', red meat and vegetable intakes. [c]Adjusted for age, residence, urban/rural status, education, 'mate/years', body mass index and red meat and vegetable intakes.

### DISCUSSION

The results of this study suggest that consumption of red meat, barbecued meat, protein, the heterocyclic amine PhIP (resulting from frying and broiling red meat) and 'mate' are associated with significant increases in the risk of RCC.

Most but not all previous studies that examined the relationship between meat intake and RCC reported an increased risk of RCC with increasing meat consumption (Maclure and Willett, 1990; McLaughlin et al, 1992; Chow et al, 1994; Wolk et al, 1996*a*). Several mechanisms have been postulated to explain this increased risk. Protein intake was suggested as the responsible factor acting through kidney damage (Chow et al, 1994). In addition, some methods of cooking red meat (e.g. frying and broiling) result in an increased amount of heterocyclic amines in meat (IARC, 1993; Wolk et al, 1996*a*). These substances are potent multiorgan mutagens and carcinogens in experimental animal studies and several reports have also suggested a role in human breast and colon cancer (De Stefani et al, 1997*a,b*). We found that a high intake of PhIP was associated with an increased risk of RCC. However, the effect of red meat was greater than the effect of protein and of PhIP, suggesting that another mechanism(s) could be responsible. More precisely, red meat is one of the major sources of total fat and saturated fat, and some studies have found an increased risk of RCC associated with saturated fat intake (Maclure and Willett, 1990; Kreiger et al, 1993). In contrast, Chow et al (1994) reported an OR of 0.6 for total fat, after adjusting for protein intake. We were unable to disentangle the effects of highly correlated variables such as red meat, protein and PhIP intakes owing to the small statistical power of our study.

Fruit intake was associated with a reduced risk of RCC in a population-based study conducted in Shanghai (McLaughlin et al, 1992), and Maclure and Willett (1990) found a protective effect of banana consumption. Unexpectedly, our results revealed an increased risk of RCC associated with fruit consumption. The variable fruits included the following individual items: orange, apple, peach, grape, pear and banana. This increased risk remained the same after controlling for red meat intake, making implausible the hypothesis that plant proteins present in the fruits could account for this result. Also, it is possible that cases had changed their fruit consumption as a result of their preclinical disease. Of course, the possibility of a chance finding cannot be ruled out.

'Mate' drinking has been suggested as a risk factor for oesophageal, oral, gastric, bladder and lung cancer in previous studies (Vassallo et al, 1985; Victora et al, 1987; De Stefani et al, 1988, 1996; De Stefani et al, 1990; Pintos et al, 1994). As 'mate' is drunk very hot, thermal injury has been postulated as the likely mechanisms in oesophageal, gastric and oral carcinogenesis (IARC, 1991). Nevertheless, the increased risk observed for bladder and lung cancer would imply other mechanisms, presumably via a chemical effect. So far no carcinogens have been detected in 'mate' (H. Barstch, personal communication; R. Adams and D. Hoffmann, personal communication). An increased risk of RCC associated with 'mate' drinking is, to our knowledge, a new finding. As 'mate' is a diuretic, a class of agents found to increase the risk of RCC, a diuretic effect could be postulated for the increased risk associated with 'mate' drinking. Finally, 'mate' contains caffeic acid (Hagiwara et al, 1991), which has been linked to kidney tumours in experimental animals, raising the possibility of a renal chemical effect. Residual confounding between 'mate' drinking and tobacco smoking has however been raised as a possibility (De Stefani et al, 1996). The effect of 'mate' drinking in increasing the risk of RCC should be further investigated and replicated in other settings.

Like most case–control studies, the present study has limitations and strengths. Firstly, the lack of information on a previous history of hypertension is a severe limitation, as this condition is related to both diet and RCC (Chow et al, 1995). Thus, hypertension is a confounder in the relationship diet–RCC and, as such, could distort the estimates observed in the study. Secondly, the statistical power of this study is limited, precluding certain detailed analyses, e.g. between variables. Also, the small size of food frequency questionnaire precluded the calculation of total energy intake and nutrients, with the exception of protein intake. Finally, the use of hospitalized controls could have masked the lack of association with tobacco smoking. A similar finding was reported in a hospital-based case–control study conducted in a French population (Benhamou et al, 1993). Among the strengths of our study, the

 © Cancer Research Campaign 1998

similar catchment area for cases and controls, the almost complete participation rate and the lack of proxy responses makes appreciable selection or classification bias unlikely.

In summary, the results of the present case–control study replicates previous findings according to which high body mass index is a major risk factor for RCC (Lindblad et al, 1994). Also, meat intake, consumption of heterocyclic amines resulting from the cooking of meat and 'mate' drinking could be associated with an increased risk of RCC in the Uruguayan population.

### REFERENCES

Baker RJ and Nelder JA (1978) *The GLIM System. Release 3*. Numerical Algorithm Group: Oxford

Benhamou S, Lenfant MH, Ori-Paoletti C and Flamant R (1993) Risk factors for renal-cell carcinoma in a French case–control study. *Int J Cancer* **55**: 32–36

Bennington JL and Laubscher FA (1968) Epidemiologic studies on carcinoma of the kidney. I. Association of renal adenocarcinoma with smoking. *Cancer* **21**: 1069–1071

Breslow NE and Day NE (1980) *Statistical Methods in Cancer Research. I. Analysis of Case–Control Studies*. IARC Scientific Publication No. 32. IARC: Lyon

Castelleto R, Castellsague X, Munoz N, Iscovich J, Chopita N and Jmelnitsky A (1994) Alcohol, tobacco, diet, mate drinking, and esophageal cancer in Argentina. *Cancer Epidemiol Biomarkers Prev* **3**: 557–564

Chow WH, Gridley G, McLaughlin JK, Mandel JS, Wacholder S, Blot WJ, Niwa S and Fraumeni JF, Jr (1994) Protein intake and risk of renal cell cancer. *J Natl Cancer Inst* **86**: 1131–1139

Chow WH, McLaughlin JK, Mandel JS, Wacholder S, Niwa S and Fraumeni JF, Jr (1995) Risk of renal cell cancer in relation to diuretics, antihypertensive drugs, and hypertension. *Cancer Epidemiol Biomarkers Prev* **4**: 327–331

Comisión Honoraria de Lucha contra el Cáncer (1993) Knowledge, beliefs, attitude and practices concerning cancer. Technical cooperation OPP/BID/PNUD. Comisión Honoraria de Lucha contra el Cáncer. Population survey. Montevideo: Comisión Honoraria de Lucha contra el Cancer, pp. 55–65 (Spanish)

De Stefani E, Correa P, Oreggia F, Leiva J, Rivero S, Fernandez G, Deneo-Pellegrini H, Zavala D and Fontham E (1987) Risk factors for laryngeal cancer. *Cancer* **60**: 3087–3091

De Stefani E, Correa P, Oreggia F, Deneo-Pellegrini H, Fernandez G, Zavala D, Carzoglio J, Leiva J, Fontham E and Rivero S (1988) Black tobacco, wine and mate in oropharyngeal cancer. *Rev Epidemiol Sante publ* **36**: 389–394

De Stefani E, Muñoz N, Esteve J, Vassallo A, Victora CG and Teuchmann S (1990) Mate drinking, alcohol, tobacco, diet, and esophageal cancer in Uruguay. A case–control study. *Cancer Res* **50**: 426–431

De Stefani E, Correa P, Fierro L, Fontham E, Chen V and Zavala D (1991) Black tobacco, mate and bladder cancer. A case–control study from Uruguay. *Cancer* **67**: 536–540

De Stefani E, Fierro L, Correa P, Fontham E, Ronco A, Larrinaga M, Balbi J and Mendilaharsu M (1996) Mate drinking and risk of lung cancer in males: a case–control study from Uruguay. *Cancer Epidemiol Biomarkers Prev* **5**: 515–519

De Stefani E, Ronco A, Mendilaharsu M, Guidobono M and Deneo-Pellegrini H (1997*a*) Meat intake, heterocyclic amines, and risk of breast cancer: a case–control study in Uruguay. *Cancer Epidemiol Biomarkers Prev* **6**: 573–581

De Stefani E, Deneo-Pellegrini H, Mendilaharsu M and Ronco A (1997*b*) Meat intake, heterocyclic amines and risk of colorectal cancer: a case–control study in Uruguay. *Int J Oncol* **10**: 573–580

FAO (1980) *Food Balance Sheets. Average 1975–77*. Food and Agriculture Organization: Rome

Hagiwara A, Hirose M, Takahashi S, Ogawa Shirai T and Ito N (1991) Forestomach and kidney carcinogenicity of caffeic acid in F344 rats and C57B1/6N * C3H/HeN F1 mice. *Cancer Res* **51**: 5655–5660

IARC (1991) *Coffee, Tea, Mate, Methylxanthines and Methylglyoxal. Monographs on the Evaluation of the Carcinogenic Risk of Chemicals to Humans*, Vol. 51. IARC: Lyon

IARC (1993) *Some Naturally Occurring Substances: Food items and Constituents, Heterocyclic aromatic amines and mycotoxins. Monographs on the Evaluation of the Carcinogenic Risk of Chemicals to Humans*. Vol. 56. IARC: Lyon

Iscovich J, Castelletto R, Esteve J, Muñoz N, Colanzi R, Coronel A, Deamezola I, Tassi V and Arslan A (1987) Tobacco smoking, occupational exposure and bladder cancer in Argentina. *Int J Cancer* **40**: 734–740

Katz DL, Zheng T, Holford TR and Flannery J (1994) Time trends in the incidence of renal carcinoma: analysis of Connecticut Tumour Registry data, 1935–1989. *Int J Cancer* **58**: 57–63

Kreiger N, Marrett LD, Dodds L, Hilditch S and Darlington GA (1993) Risk factors for renal cell carcinoma: results of a population-based case–control study. *Cancer Causes Control* **4**: 101–110

Lindblad P, McLaughlin JK, Mellengaard A and Adami H-O (1993) Risk of kidney cancer among patients using analgesics and diuretics: a population-based cohort study. *Int J Cancer* **55**: 5–9

Lindblad P, Wolk A, Bergstrom R, Persson I and Adami HO (1994) The role of obesity and weight fluctuations in the etiology of renal cell cancer: a population-based case–control study. *Cancer Epidemiol Biomark Prev* **3**: 631–639

Maclure M and Willett W (1990) A case–control study of diet and risk of renal adenocarcinoma. *Epidemiology* **1**: 430–440

Mazzei ME and Puchulu MR (1991) *Table of Chemical Composition of Foods*. Cenexa: Buenos Aires (in Spanish)

McLaughlin JK, Gao IT, Gao RN, Zheng W, Ji BT, Blot WJ and Fraumeni JF, Jr (1992) Risk factors for renal-cell cancer in Shanghai, China. *Int J Cancer* **52**: 562–565

McLaughlin JK, Lindblad P, Mellemgaard A, McCredie M, Mandel JS, Schlehofer B, Pommer W and Adami HO (1995) International renal-cell cancer study. I. Tobacco use. *Int J Cancer* **60**: 194–198

Parkin DM, Whelan S, Ferlay J, Raymond L and Young J (1997) *Cancer Incidence in Five Continents*, Vol. VII (IARC Scientific Publications No. 143). IARC: Lyon

Pintos J, Franco EL, Oliveira BV, Kowalski LP, Curado MP and Dewar R (1994) Mate, coffee, and tea consumption and risk of cancers of the upper aerodigestive tract in Southern Brazil. *Epidemiology* **5**: 583–590

Talamini R, Baron AE, Barra S and La Vecchia C (1990) A case–control study of risk factors for renal cell cancer in northern Italy. *Cancer Causes Control* **1**: 125–131

Vassallo A, De Stefani E, Correa P, Cendan M, Zavala D, Chen V, Carzoglio J and Deneo-Pellegrini H (1985) Esophageal cancer in Uruguay. A case–control study. *J Natl Cancer Inst* **75**: 1005–1009

Victora CG, Muñoz N, Day NE, Barcelos LB, Peccin DA and Braga NM (1987) Hot beverages and oesophageal cancer in Southern Brazil: a case–control study. *Int J Cancer* **39**: 710–716

Weinmann S, Glass AG, Weiss NS, Psati BM, Siscovick DS and White E (1994) Use of diuretics and other antihypertensive medication in relation to the risk of renal cell cancer. *Am J Epidemiol* **140**: 792–804

Wolk A, Gridley G, Niwa S, Lindblad P, McCreadie M, Mellemgaard A, Mandel JS, Wahrendorf J, McLaughlin JK and Adami H-O (1996*a*) International renal cell cancer study. VII. Role of diet. *Int J Cancer* **65**: 67–73

Wolk A, Lindblad P and Adami H-O (1996*b*) Nutrition and renal cell cancer. *Cancer Causes Control* **7**: 5–18

Wynder EL, Mabuchi K and Whitmore WF (1974) Epidemiology of adenocarcinoma of the kidney. *J Natl Cancer Inst* **53**: 1619–1634

© Cancer Research Campaign 1998